# Interleukin-21 modulates balance between regulatory T cells and T-helper 17 cells in chronic hepatitis B virus infection

**DOI:** 10.1186/s12879-023-08723-w

**Published:** 2023-10-24

**Authors:** Yun Cai, Hailei Ji, Xin Zhou, Kai Zhao, Xiaoping Zhang, Liang Pan, Ruihua Shi

**Affiliations:** 1 Medical School, Southeast University, 87 Dingjiaqiao Road, Nanjing, 210009 China; 2 Department of Gastroenterology Disease, The First People’s Hospital of Jintan District, Changzhou, 213200 China; 3 Department of Infections Disease, The Third People’s Hospital of Zhenjiang, Zhenjiang, 212000 China; 4 Department of Gastroenterology Disease, The Affiliated Zhongda Hospital of Southeast University, Nanjing, 210009 China

**Keywords:** Chronic Hepatitis B virus Infection, Th17 cells, Treg cells, IL-21, STAT3

## Abstract

**Background:**

Chronic HBV infection is always accompanied by differences in the balance between regulatory T cells (Tregs) and T-helper 17 (Th17) cells in infection phases. IL-21 plays an important role in the progression of chronic HBV infection. Thus, the aim of our study was to investigate the role of the regulatory function of IL-21 in maintaining the balance between Tregs and Th17 cells in chronic HBV infection.

**Methods:**

Twenty-five chronic HBV-infected patients in the immune-tolerant (IT) phase and 23 chronic hepatitis B (CHB) patients were recruited in this study. Cytokines production was measured by ELISA. The mRNA expression levels were determined by qPCR. CD4^+^T cells were stimulated with or without IL-21. Tregs and Th17 cells were measured by flow cytometry. pSTAT3 and STAT3 expression was assessed by Western blotting.

**Results:**

The concentration of IL-21 in the serum of CHB were significantly higher than that in the serum from IT patients, and IL-21 and IL-21R levels in the PBMCs from CHB were higher than those from IT patients. IL-21 promoted Th17 cells differentiation and function but inhibited Treg cells differentiation and function by activating STAT3 signaling pathways, upregulating RORγt expression, downregulating Foxp3 expression, by increasing IL-17and IL-22 secretion, and decreasing TGF-β secretion in chronic HBV infection. The proportion of Tregs and TGF-β concentrations in CHB was significantly lower than that in IT patients. Furthermore, the percentage of Th17 cells and the IL-17 concentration in CHB was markedly higher than that in IT patients, causing a reduction in the Tregs/Th17 ratio in CHB patients.

**Conclusions:**

Our results suggest that IL-21 may contribute to inflammation in chronic HBV infection by modulating the balance between Treg and Th17 cells.

**Supplementary Information:**

The online version contains supplementary material available at 10.1186/s12879-023-08723-w.

## Background

Chronic hepatitis B virus (HBV) infection is a serious public health problem worldwide. Recent World Health Organization (WHO) estimates show that the number of patients with persistent infections is 350 million [1, 2]. In many patients, HBV infection progresses to end-stage liver disease, including liver failure, liver cirrhosis, and hepatocellular carcinoma [3]. It is well known that the chronic HBV infection is a dynamic process reflecting the interaction between the host immune system and virus replication [4]. In 2017, the European Association for the Study of the Liver divided the natural history of chronic HBV infection into five phases, considering the presence of HBeAg, HBV DNA levels, alanine aminotransferase (ALT) values, and liver inflammation [5]. HBeAg-positive chronic HBV infection has been divided into two phases: chronic HBV infection in the immune-tolerant(IT) phase and chronic hepatitis B(CHB). CHB may occur after several years of chronic HBV infection or more rapidly in subjects infected during adulthood. However, the mechanism of the transition from chronic HBV infection in the IT phase to CHB is not fully understood.

Chronic HBV infection induces some specific T-cell responses, which play an important role for viral clearance and infection control [6].CD4^+^ T cells can differentiate into multi-specific subtypes, which have pivotal functions in humoral and cellular immunity. Regulatory T cells (Tregs) are a subset of CD4 ^+^ T cell effectively inhibit immune cells by secreting cytokines IL-10 and TGF-β, to mediate the immune tolerance and maintain the immune balance, thereby controlling liver inflammation and improving liver pathology. Nevertheless, it may exert adverse effects on HBV clearance [7, 8]. Th17 cells, another subset of CD4^+^ T cells, secrete IL-17, associated with liver inflammation and fibrosis [9, 10].Although Treg and Th17 cells are both differentiated from CD4^+^ T cells, they are mutually transformed during their differentiation and have mutually restricting functions. Thus, Treg/Th17 balance plays a crucial role in the progression of chronic HBV infection [11, 12].

Interleukin-21 (IL-21) is a type I cytokine produced by a number of CD4^+^ T cell subsets, with the highest production by Th17 cells and follicular helper T (Tfh) cells. IL-21 sustains or promotes Tfh and Th17 cells through STAT3 activation in an autocrine fashion [13–15]. IL-21 mediates a variety of biological immune responses through effector cells involved in the immune network by activating STAT3 signaling pathways [16–18]. IL-21 was found to modulate the balance betweenTh17 and Treg cells in several diseases [19, 20]. Previous studies evidenced that serum IL-21 levels are elevated in chronic hepatitis B [21, 22]. Nonetheless, the effect of IL-21 on Tregs/Th17 cells balance during the progression of chronic HBV infection has not yet been elucidated. Thus, in the present study, we investigated the circulating IL-21 levels and Treg and Th17 cell populations. Additionally, we explored the mechanism of the effect of IL-21 on Tregs/Th17 function in patients with chronic HBV infection.

## Methods

### Enrolled patients

A number of 25 chronic HBV-infected patients in the IT phase and 23 chronic hepatitis B (CHB) patients were recruited in this study. All patients were enrolled in The First People’s Hospital of Jintan District, Changzhou, and The Third People’s Hospital of Zhenjiang (Jiangsu, China) from November 2021 to December 2022. These patients were diagnosed incompliance with the diagnostic standard of Chinese Guideline of Prevention and Treatment for Chronic Hepatitis B (2019 Version). All patients were HBV e antigen(HBeAg)-positive. Their HBV-DNA load ≥ 5 lg copies/mL, serum alanine aminotransferase (ALT) level ≥ twice the upper limit of the normal level in CHB patients, while the ALT level was normal in the chronic HBV-infected patients in the IT phase. Patients coinfected with human immunodeficiency virus, hepatitis A, C, D, and other potential causes of chronic liver damage, such as alcohol, drugs, and autoimmune diseases, were excluded from the study. No patients received immunotherapy. Samples, each containing 20mL of heparinized blood, were then collected. Next, 4mL of serum was obtained from the subjects for measurement of cytokines concentrations and stored at -80℃. This study was approved by the Ethics Committee of The First People’s Hospital of Jintan District, Changzhou. Signed informed consent was obtained from all participants before the study. The clinical parameters of the enrolled patients are presented in Table 1.

**Table 1:** Clinical parameters of the enrolled patients

Group	Chronic HBV infection in the Immune-tolerant phase	Chronic hepatitis B
Case	25	23
Sex (male/female)	11/14	11/12
Age (years)	28.50 ± 10.02	33.20 ± 11.09
ALT (U/L)	16.40 ± 2.56	248.18 ± 88.45
HBV-DNA(lg copies/mL)	7.56 ± 0.82	6.86 ± 0.71
HBsAg positive	25	23
HBeAg positive	25	23
Anti-HBs positive	0	0
Anti-HBe positive	0	0
Anti-HBc positive	25	23

Data are expressed as mean ± standard deviation (SD).

### Virological and biochemical assessments

Serum HBV DNA was quantified by fluorescent quantitative PCR with commercially available kits (PE/B/MJ/L, Shenzhen, China) with a detection limit of 500 IU/mL. HBV surface antigen (HBsAg), HBeAg, anti-HBe, anti-HBs, and anti-HBc was measured by commercial enzyme-linked immunosorbent assay (ELISA) kits (Kehua Biotech, Shanghai, China). Serum ALT levels were measured with an automatic biochemical analyzer (7500, Hitachi, Tokyo, Japan).

### Enzyme-linked immunosorbent assays (ELISA)

IL-17, IL-22, IL-10, and TGF-β concentrations in the supernatant of CD4^+^ T cells, and IL-21, IL-17, IL-22, and TGF-β concentrations in the serum, were measured using commercial ELISA kits (R&D Systems, Minneapolis, MN, USA) following the manufacturer’s instructions.

### CD4^+^ T cell purification and culture

First, PBMCs were isolated from heparinized blood by density gradient centrifugation using Ficoll-Hypaque (Sigma-Aldrich, MO, USA). The CD4^+^ T cells were purified from the isolated PBMCs using CD4^+^ T Cell Isolation Kit (Miltenyi, Bergisch Gladbach, Germany). The purity of CD4^+^ T cells, determined by flow cytometry, was more than 98%. The purified CD4^+^T cells were cultivated at a density of 5 × 10^5^ cells/well in RPMI 1640 medium, supplemented with 10% fetal bovine serum, 1 µg/mL anti-CD28, 1 µg/ml anti-CD3 and 30 U/Ml rIl-2 with or without 50 ng/mL IL-21 for 72 h.

### Flow cytometric analysis and intracellular staining

The following antibodies with their isotype controls were purchased from Biolegend: anti-CD4-BV510, anti-CD3-APC-cy7, anti-IL-17-PE, anti-CD45-PE-cy5.5, anti-IL-21-PE, Anti-IL-21R-PE, anti-CD25-BV421, and anti-Foxp3-AF488. For intracellular staining, the cells were incubated with R10 medium, which is constituted by RPMI1640, 100 ug/mL streptomycin, 100 U/mL penicillin, and 2 mm glutamine, coupled with 50 ng/mL phorbolmyristate acetate (PMA, Sigma, USA), 1 µg/mL ionomycin (Sigma, USA) for 2 h; then 0.7 µL/mL GolgiStop™(BD Biosciences, USA) was added to the cells and incubated for another 3 h. For surface staining, the cells were washed with staining buffer and incubated with fluorescence-conjugated antibodies for 15 min. Then, the cells were washed two times with staining buffer, fixed with fixation buffer for 20 min, permeabilized, and stained with fluorescence-conjugated antibodies for 20 min. The data acquired using a FACS Canto-II flow cytometer (BD Biosciences) were analyzed with Flow Jo software (Tree Star).

### Real-time quantitative PCR (qPCR)

Total RNA was isolated from PBMCs using Trizol reagent (Invitrogen, CA, USA) and reverse-transcribed with PrimeScript RT Reagent Kit (TaKaRa, Beijing, China) following the manufacturer’s instructions. The primer sequences were as follows: β-actin (FW 5’-TGG CAC CCA GCA CAA TGA A-3’, RV 5’-TAA GTC ATA GTC CGC CTA GAA GCA-3’), IL-17(FW5’-CTG AAC ATC CAT AAC CGG AAT ACC A-3’, RV 5’-AGC GTT GAT GCA GCC CAA G-3’), IL-22 (FW 5′-CCA GGC TCA GCA ACA GGC TAA-3′, RV 5′-TTT CAG CTT TGC TCT GGT CAA ATG-3’), IL-10 (FW5’-CAA GAC CCA GAC ATC AAG GCG-3′, RV 5′- GCA TTC TTC ACC TGC TCC ACG-3’),TGF-β (FW 5’-CGC GTG CTA ATG GTG GAA A-3’, RV 5’-CGC TTC TCG GAG CTC TGA G-3’), (IL-21 (FW 5’-CCA AGG TCA AGA TCG CCA CA-3’, RV 5’-TTC TGG AGC TGG CAG AAA TTC A-3’), IL-21R (FW 5′-AGA CCC TCA ATA AAC GTC AGC TTC C-3′, RV 5′-TCG CTG ACG ATT GAT GTT CTC AC-3′), RORγt (FW 5’-GCT GTG ATC TTG CCC AGA ACC-3’, RV 5’-TGC CCA TCA TCA TTG CTG TTA ATC C-3’), FoxP3 (FW 5′-CCT CCC CCA TCA TAT CCT TT-3′, RV 5′-TTG GGG TTT GTG TTG AGT GA-3′). Fluorescent quantitative PCR instrument(CFX 96 Real-Time System, Bio-RAD, USA) was used and the reaction system (20 µL) consisted of 10µL of 2× Ultra SYBR mixture, 0.4 µL of Primer F/R, 1 µL of cDNA, and RNase-free water up added to 20 µL. qPCR was performed under the following conditions: pre-denaturation at 95℃ for 10 min, denaturation at 95 ℃ for 15 s, annealing and extension at 60℃ for 1 min; a total number of 40 cycles was performed. Then, dissolution curve analysis was conducted at 95 ℃ for 15s; 60 ℃ for 1 min; 95 ℃ for 15 s; and 60℃ for 15s. The relative gene expression was quantified by the 2^−ΔΔCT^ method.

### Western blot

Cells were lysed in SDS buffer with β-mercaptoethanol for 5 min and incubated at a 95 °C for 10 min. The supernatants were collected by centrifugation for 1 min at 10,000×g. Total proteins were subjected to SDS-PAGE gel electrophoresis and transferred onto PVDF membranes. Then, the membranes were incubated overnight with rabbit polyclonal antibodies to signal transducers and activators of transcription 3 (STAT3, phospho Y705, ab267373) or STAT3 (ab68153) (Abcam, Cambridge, MA, USA; 1: 1000 dilution). Horseradish peroxidase-conjugated goat anti-rabbit antibody IgG (Abcam; Cambridge, MA, USA; 1: 2000 dilution) was added incubating for additional 2 h. The protein bands were observed with an Odyssey Infrared Imaging System (LICOR, USA).

### Statistical analyses

All data were analyzed using SPSS Version 23.0. Data are presented as mean ± standard deviation (SD). The statistical differences between the two groups were determined by two-tailed unpaired or paired Student’s *t-*test. Spearman’s rank-order correlation coefficient was employed to evaluate the correlations. All statistical analyses hypothesis tests were performed at a significance level of *P* < 0.05.

## Results

### IL-21 and IL-21R expression levels are increased in CHB patients

**Fig. 1:**
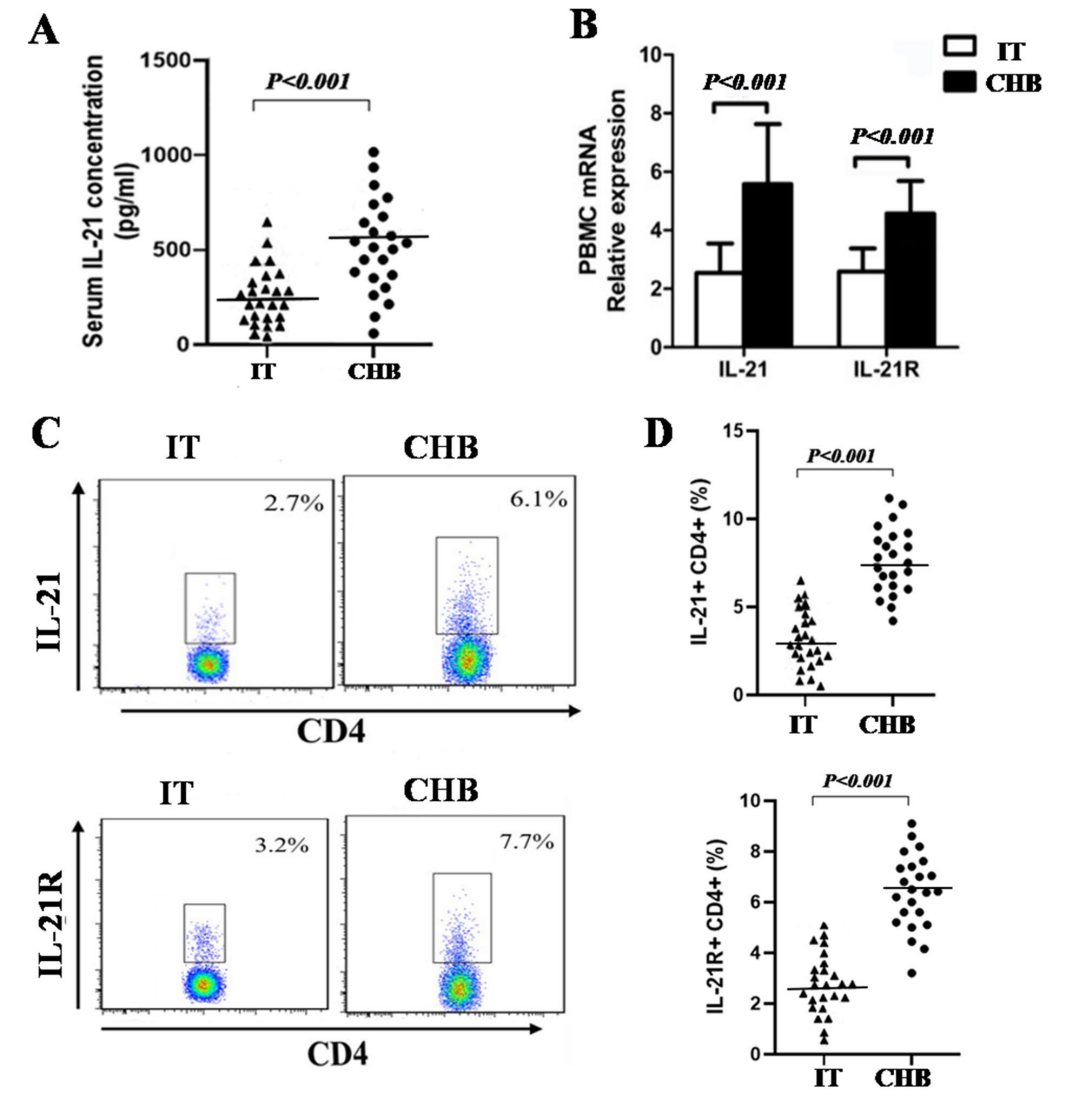
IL-21 and IL-21R expression in the peripheral blood of chronic HBV- infected patients in the immune-tolerant (IT) phase (n = 25) and chronic hepatitis B (CHB) patients (n = 23). (**A**) The concentration of IL-21 in the serum of IT and CHB patients. (**B**) IL-21 and IL-21R mRNA expression in PBMCs of IT and CHB patients. (**C**–**D**) Flow cytometry of IL-21 and IL-21R expression in CD4^+^ T cells from IT and CHB patients. Data show means ± SD. *P*-values were tested using unpaired Student’s t-test

Our research results revealed that the concentration of IL-21 in the serum of CHB were significantly higher than that in the serum from IT patients (*P* < 0.001, Fig. 1A). Furthermore, the mRNA expression of IL-21 and IL-21R was significantly higher in the PBMCs from CHB patients than in those from IT patients (*P* < 0.001, Fig. 1B). As IL-21 is secreted mainly by CD4^+^ T cells, we assessed IL-21 secretion by the CD4^+^ T cells in PBMCs of CHB and IT patients. Our results showed that the percentage of CD4^+^ T cells secreting IL-21 and expressing IL-21R in the PBMCs in CHB patients was significantly higher than that in IT patients(*P* < 0.001, Fig. 1C–D).Overall, IL-21 and its receptor were markedly higher in the CD4^+^ T cells from PBMCs from CHB patients than those from IT patients.

### IL-21 promotes Th17 cell differentiation and inhibits Treg cell differentiation

**Fig. 2:**
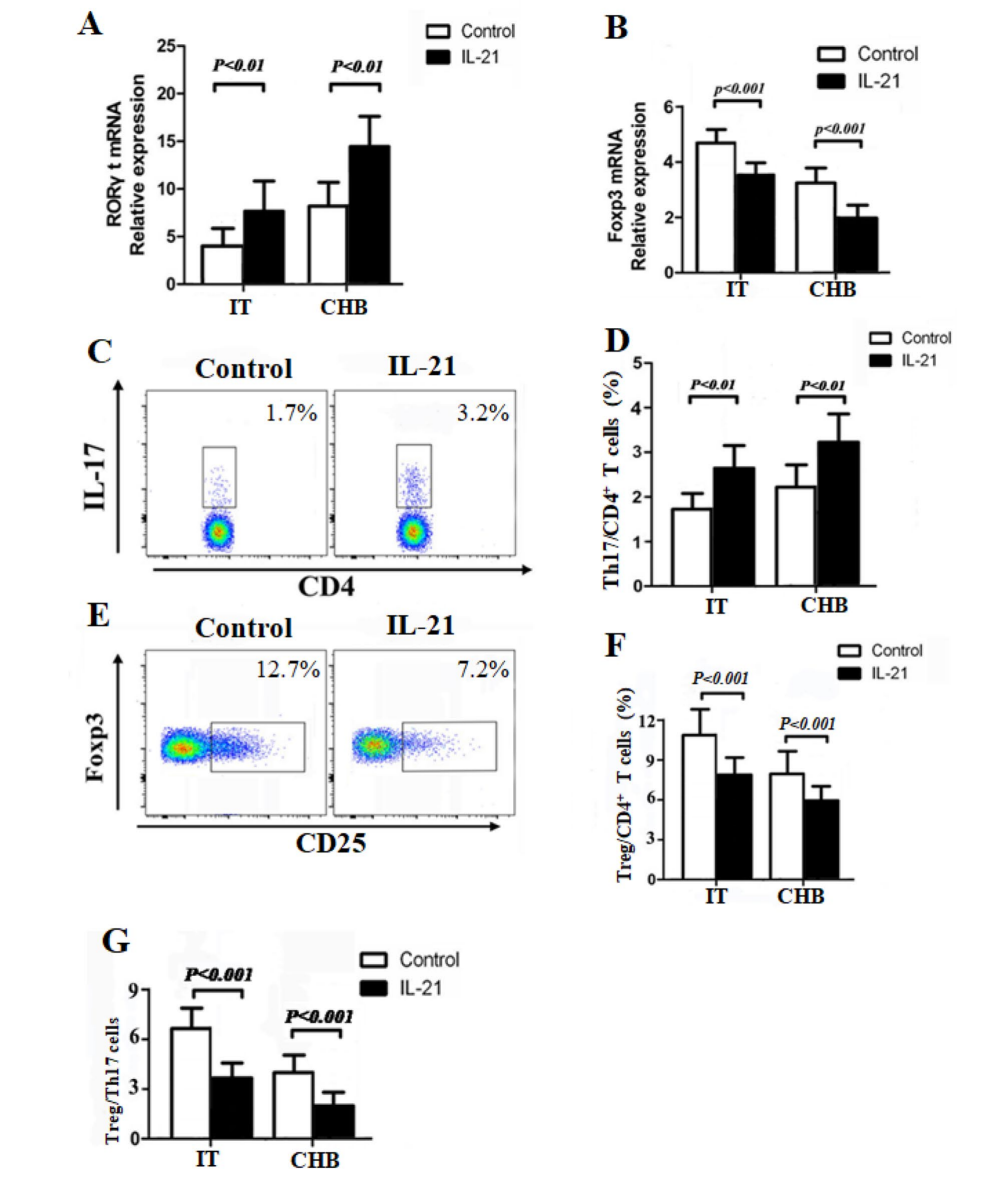
IL-21 promotes Th17 cell differentiation and inhibits Treg cell differentiation in chronic HBV-infected patients in the immune-tolerant (IT) phase (n = 25) and chronic hepatitis B (CHB) (n = 23) patients. (**A**–**B**) RORγt and Foxp3 mRNA expression in IT and CHB patients with or without IL-21 stimulation. (**C**-**D**) The proportion of Th17 cells in CD4^+^ T cells from IT and CHB patients with or without IL-21 stimulation. (**E**–**F**) The proportion of Treg cells in CD4^+^ T cells from IT and CHB patients with or without stimulation IL-21. (**G**) Ratio of Tregs/Th17 in IT and CHB patients with or without IL-21 stimulation. Data show means ± SD. P-values were tested using paired Student’s *t*-test

We hypothesized that IL-21 also have effects on the Treg/Th17 cells balance during chronic HBV infection. Our present study showed that the mRNA expression of RORγt in CD4^+^ T cells was significantly increased after IL-21 stimulation both in IT and CHB patients (*P* < 0.01, Fig. 2A), which plays a critical role in the differentiation of Th17 cells. Thus, we measured the levels of Th17 cells by flow cytometry and found that the proportion of Th17 cells in the CD4^+^ T cells was increased after stimulation with IL-21 in both IT and CHB patients (*P* < 0.01, Fig. 2 C, D). However, the expression of Foxp3 in CD4^+^ T cells was reduced after the treatment with IL-21 both in IT and CHB patients (*P* < 0.001, Fig. 2B), which is essential for the differentiation and function of Tregs. In addition, the frequency of Tregs was notably decreased in response to IL-21 stimulation in both IT and CHB patients(*P* < 0.001, Fig. 2E, F), leading to a subsequent decrease in the ratio Tregs/Th17 after stimulation with IL-21 in both IT and CHB patients (*P* < 0.001, Fig. 2G). Overall, these results revealed that IL-21 promoted Th17 cell differentiation but inhibited Treg cell differentiation by upregulating RORγt expression and downregulating Foxp3 expression.

### IL-21 promotesTh17-related cytokines expression and regulates Treg-related cytokines expression

**Fig. 3:**
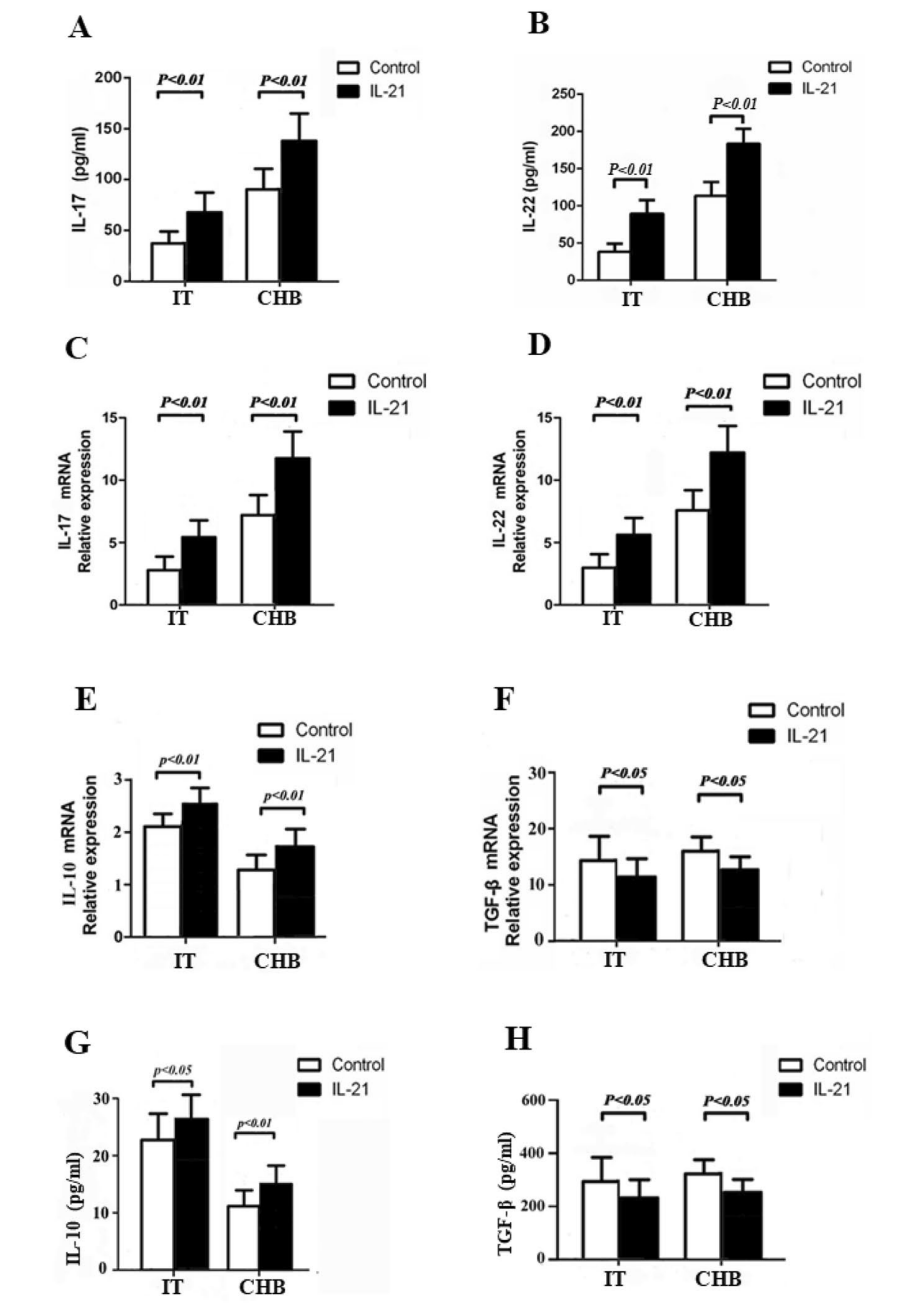
IL-21 promotes Th17-related cytokines expression and regulates Treg-related cytokines expression in chronic HBV-infected patients in the immune-tolerant (IT) phase (n = 25) and chronic hepatitis B (CHB) (n = 23) patients. (**A**–**B**) IL-17 and IL-22 expression in cultured supernatants from IT and CHB patients with or without IL-21 stimulation. (**C**–**D**) IL-17 and IL-22 mRNA expression from IT and CHB patients with or without IL-21 stimulation. (**E**–**F**) IL-10 and TGF-β mRNA expression from IT and CHB patients with or without IL-21 stimulation. (**G**–**H**) IL-10 and TGF-β expression in cultured supernatants from IT and CHB patients with or without IL-21 stimulation. Data show means ± SD. *P*-values were tested using paired Student’s *t*-test

As known, Th17 cells secrete mainly IL-17 and IL-22 to perform their functions, contributing to the progression of chronic hepatitis B virus infection. Our results showed that IL-17 and IL-22 culture concentration and mRNA expression were notably elevated after IL-21 stimulation in both IT and CHB patients (*P* < 0.01, Fig. 3A–D). IL-10 and TGF-β are key cytokines secreted by Treg cells that contribute to their immunosuppressive function. We established that TGF-β concentration and mRNA expression were reduced significantly after IL-21 treatment in both IT and CHB patients (*P* < 0.05, Fig. 3F, H).However, the concentration of IL-10 and mRNA expression were markedly increased after IL-21 stimulation in IT and CHB patients (*P* < 0.01, Fig. 3E,G).This effect maybe due to a mechanism triggered to compensate for the decline in Treg cell proportions after IL-21 stimulation. In summary, our results confirm that IL-21 promotes Th17-related cytokines expression and regulates Treg-related cytokines expression in chronic HBV infection.

### IL-21 modulates the balance between Treg and Th17 cells by activating STAT3 signaling pathways

**Fig. 4:**
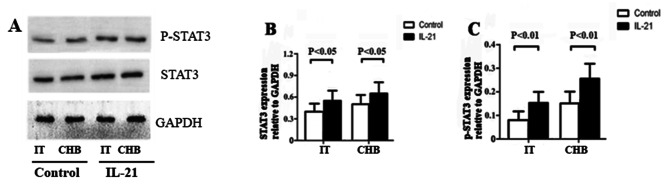
IL-21 activates STAT3 signaling pathways in chronic HBV-infected patients in the immune-tolerant (IT) phase (n = 25) and chronic hepatitis B (CHB) (n = 23) patients. (**A**) phosphorylated STAT3 (pSTAT3), total STAT3 expression in CD4^+^ T cells from IT and CHB patients with or without IL-21 stimulation were tested by Western blot. (**B**) STAT3 expression relatives to GAPDH were comparable between IT and CHB patients. (**C**) pSTAT3 expression relatives to GAPDH were comparable between IT and CHB patients. Data show means ± SD. *P*-values were tested using paired Student’s *t*-test

IL-21 activates JAK-STAT, MAP kinase (MAPK), and PI3-kinase (PI3K) pathways; STAT3 plays a major role in a variety of biological actions of IL-21 [23]. STAT3 phosphorylation (pSTAT3) and total STAT3 expression in CD4^+^ T cells of IT and CHB patients with or without IL-21 stimulation was assessed by Western blot. IL-21 stimulation resulted in significantly upregulated pSTAT3 and total STAT3 expression in cultured CD4 + T cells from IT and CHB patients (*P* < 0.05, Fig. 4A–C). Overall, our results revealed that the IL-21 promoted Th17 cell differentiation and inhibited Treg cell differentiation, leading to an imbalance between Treg and Th17 cells by activating STAT3 signaling pathways in patients with chronic HBV infection.

### Imbalance in Th17 and Treg cells populations from IT and CHB patients

**Fig. 5:**
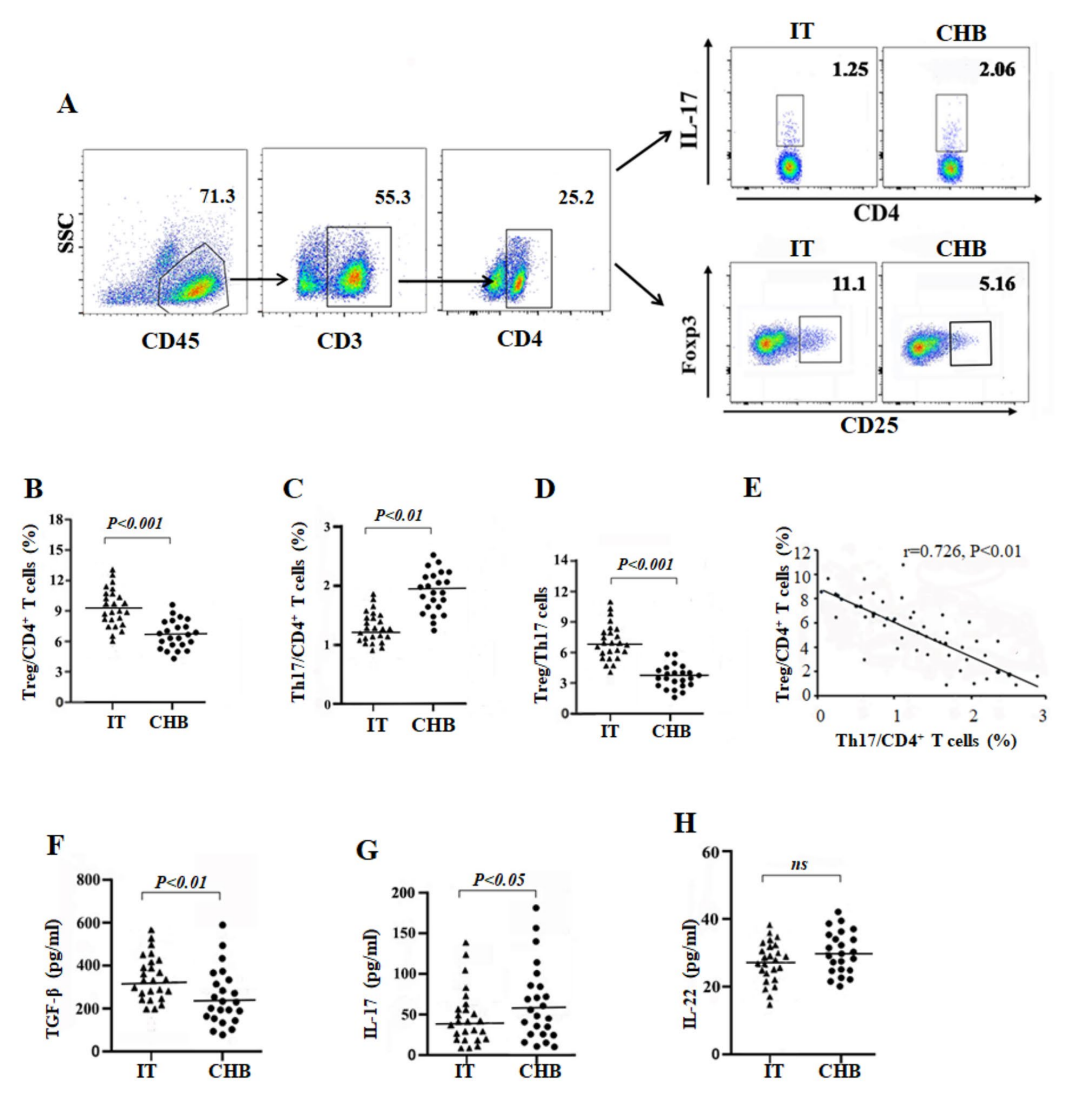
The proportion of regulatory T cells (Tregs) and T-helper 17 (Th17) cells in chronic HBV-infected patients in the immune-tolerant (IT) phase (n = 25) and chronic hepatitis B (CHB) (n = 23) patients. (**A**) Gating strategy and representative flow dots for Tregs and Th17 cells in both IT and CHB patients. The CD4^+^ T cell staining was performed with antibodies against CD45, CD3, and CD4, then the staining CD4^+^ T cell with antibodies against CD25, FOXP3, IL-17, the cells CD4 + CD25 + FOXP3 + are considered to be Tregs, and the cells CD4 + IL-17 + are considered to be Th17 cells. (**B**) Tregs percentage in CD4^+^ T cells was comparable between IT and CHB patients. (**C**) Th17 cells percentage in CD4^+^ T cells was comparable between IT and CHB patients. (**D**) Ratio of Treg/Th17 cells was comparable between IT and CHB patients. (**E**) Correlation between the percentage of Tregs and Th17 cells of all subjects (n = 48). (**F**–**H**) The concentration of TGF-β, IL-17, and IL-22 in the serum of IT and CHB patients. Data show means ± SD. *P*-values were tested using unpaired Student’s *t*-test

In the present study, we measured the percentages of Th17 and Treg cells in PBMCs from IT and CHB (Fig. 5A).The proportion of Tregs in CHB was notably lower than that in IT patients(*P* < 0.001, Fig. 5B), and the percentage of Th17 cells in CHB was notably higher than that in IT patients(*P* < 0.001, Fig. 5C), leading to a significantly lower ratio Tregs/Th17 in CHB than in IT patients(*P* < 0.001, Fig. 5D). Furthermore, our research also showed that the frequency of Treg cells was negatively correlated with that of Th17 cells in all participants (*P* < 0.01, Fig. 5E). Our results confirmed that IL-21 was more highly expressed in CHB patients than in IT patients, and promoted the cell differentiation of Th17 while inhibiting that of Treg. Thus, the increased Th17 cells and decreased Tregs in CHB patients may be due to the upregulated expression of IL-21 in CHB as compared with that in IT patients. Therefore, IL-21 may play a crucial role in the progression from the IT phase to the chronic hepatitis B phase of chronic HBV infection. In addition, we found that the concentration of TGF-β in the serum of IT patients was significantly higher than that in the serum of CHB patients (P < 0.01, Fig. 5F), and the concentration of IL-17 was significantly lower in the serum of IT patients than in that of CHB patients (P < 0.05, Fig. 5G), However, no significant difference was observed in IL-22 concentration between the two groups (Fig. 5H). Altogether, we established that IL-21 promotes the progression of chronic HBV infection by regulating the balance between Th17 and Treg cell populations and their functional mediators IL-17 and TGF-β.

## Discussion

IL-21 action is associated with immune injury and viral replication in infectious diseases [24, 25]. Previous studies have shown that the increase in IL-21 levels in CHB patients is accompanied by an rise in those of Th17 cells, and that IL-21 concentrationis positively associated with the degree of liver inflammation and injury in patients with chronic HBV infection [21, 22]. Consistent with the results of previous studies, our findings evidenced that the IL-21 level in the peripheral blood of CHB patients was significantly higher than that in the peripheral blood of chronic HBV infected patients in the IT phase. In addition, our results revealed that the percentage of CD4^+^ T cells secrete IL-21 and express IL-21R was notably increased in the PBMCs of CHB patients compared with IT patients, suggesting that the CHB patients may have more pronounced liver inflammation and injury due to the upregulated expression of IL-21. Addition, our results are consistent with the characteristics of ALT ≥ twice the upper limit of the normal level in patients with chronic hepatitis and ALT level was normal in chronic HBV-infected patients in the IT phase, suggesting that the information of CHB patients are more compared with IT patients.

As IL-21 have reported multiple effects on the balance between Treg and Th17 cells in several diseases [19, 20], we then hypothesized that IL-21 may also have effects on Treg/Th17 cells balance in chronic HBV infection. Our in vitro experiment demonstrated that IL-21 promoted CD4^+^ T cell from the PBMCs differentiation into Th17 cells by upregulating the expression of RORγt both in CHB and IT patients, and inhibited the differentiation of Treg cells by downregulating the expression of Foxp3 both in CHB and IT patients. IL-21 also enhanced the function of Th17 cells by increasing secretion cytokines IL-17 and IL-22, and inhibited the function of Tregs by reducing secretion cytokines TGF-β. However, the culture supernatant level of Treg-secreted cytokine IL-10 was markedly increased after IL-21 stimulation, which might represent a compensatory mechanism triggered by the decline in Tregs frequency. IL-21 activates the JAK-STAT, MAP kinase (MAPK), and PI3-kinase (PI3K) pathways, and STAT3 plays a major role in a variety of biological actions of IL-21 [23].We found that IL-21 increased the expression of phosphorylated STAT3 and STAT3 in cultured CD4^+^ T cells in CHB and IT patients, which were assessed by Western blot, which is consistent with previous findings that IL-21 sustains or promotes Th17 cell differentiation through STAT3 activation in an autocrine fashion [26, 27].The STAT protein is involved JAK(Janus kinase)-STAT signaling pathways, which is involved in a range of biological responses in the human body, such as inflammation, immunity reactions, apoptosis and proliferation, as well as viral infection, liver fibrosis, and liver cancer [28].

In a previous investigation, Yang found significantly decreased RORγt expression but increased FOXP3 expression in STAT3-deficient T-helper cells, suggesting that STAT3 is required for RORγt expression during Th17 differentiation, and STAT3 might inhibit the induction of Treg differentiation [29]. Overall, our results raise the possibility that IL-21 enhances Th17 cells differentiation and function but inhibits Treg cells differentiation and function. This action is performed by activating STAT3 signaling pathways, upregulating RORγt expression, downregulating Foxp3 expression, increased IL-17 and IL-22 secretion, and decreased TGF-β secretion in chronic HBV infection patients.

Earlier evidence has shown that the percentage of Treg cells is correlated with immune tolerance and control immune response in chronic HBV infection [30–33]. The frequency of Tregs was found to increase in IT patients, which reduced the damage to the liver cells but also delayed the clearance of HBV, leading to chronic disease [34, 35].We found that the percentage of Treg cells and secretive cytokine TGF-β concentration was higher in IT compared with CHB patients, suggesting a greater immune tolerance and delayed clearance of HBV in this phase, which had been previously termed the ‘immune tolerant’ phase in natural history of chronic HBV infection.

Then, the second phase in the natural history of chronic HBV infection is HBeAg-positive CHB, according to EASL 2017 Clinical Practice Guidelines on the management of hepatitis B virus infection. Previous studies [36, 37]revealed that Th17 cells could induce inflammatory response and liver injury in CHB patients, and the frequency of Th17 cells was associated with higher levels of total bilirubin (TB) and direct bilirubin (DB) in patients with CHB [38, 39]. In the present study, the percentage of Th17 cells and IL-17 concentration in CHB was notably higher than those in IT patients, which indicates a more pronounced inflammatory response in CHB patients. As known, the imbalance between Tregs and Th17 cells is associated with chronic HBV infection [40, 41].Our results showed that the ratio of Tregs/Th17 in CHB patients was significantly lower than that in IT patients. Moreover, the frequency of Treg cells was negatively correlated with that of Th17 cells in both CHB and IT patients. We also found that IL-21 levels were significantly higher in the peripheral blood of CHB patients than in that of IT patients. Next, we confirmed that IL-21 enhanced Th17 differentiation but inhibited that of Treg cells. Herein, the changes in the proportions and functions of Tregs and Th17 cells in PBMCs may be due to the different expression levels of IL-21 in CHB and IT patients. Overall, IL-21 may play a significant role in the progression of from the immune-tolerant to the immune-active phase of CHB. Therefore, IL-21 may drive chronic HBV infection from the IT phase into chronic hepatitis B phase by regulating the balance of Th17 and Treg cells and their functional mediators in chronic HBV infection.

## Conclusions

In summary, our research findings show that IL-21 contributes to the progression of chronic HBV infection. This action is performed by enhancing Th17 cells differentiation and function but inhibiting Treg cells differentiation and function by activating STAT3 signaling pathways, upregulating RORγt expression, downregulating Foxp3 expression, increasing the secretion of IL-17, and decreasing the secretion of TGF-β. Therefore, IL-21 may serve as a potential therapeutic target in chronic HBV infection.

### Electronic supplementary material

Below is the link to the electronic supplementary material.

Supplementary Material 1

## Data Availability

All data generated or analyzed during this study are included in this article.

## List of Abbreviations

HBV: Hepatitis B virus
CHB: Chronic hepatitis B
IL-21: Interleukin-21
ELISA: Enzyme-linked immunosorbent assay
qPCR: Quantitative polymerase chain reaction
PBMCs: Peripheral blood mononuclear cells
Th17: T-helper17
