# Supplementary figures and images for: Interleukin-21 modulates balance between regulatory T cells and T-helper 17 cells in chronic hepatitis B virus infection
Source: BMC Infect Dis. 2023 Oct 24;23:719. doi: 10.1186/s12879-023-08723-w (PMC10594809; doi:10.1186/s12879-023-08723-w)

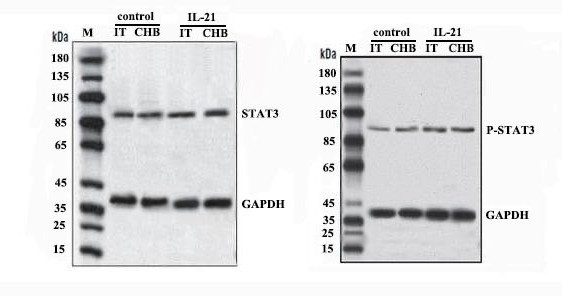

Supplement: Supplementary file 1 — Supplementary Material 1 [file 12879_2023_8723_MOESM1_ESM.png]
